# Development and validation of an Arabic tool for assessment of post-vaccination confidence in COVID-19 vaccines (ARAB-VAX-CONF)

**DOI:** 10.1186/s42506-024-00174-8

**Published:** 2024-11-18

**Authors:** Rowan Abuyadek, Samar Abd ElHafeez, Mohamed Mostafa Tahoun, Sally Samir Othman, Abdelrahman Omran, Naglaa Fathy, Ramy Mohamed Ghazy

**Affiliations:** 1 Health Administration and Behavioural Sciences Department, High Institute of Public Health, Alexandria University, Alexandria, Egypt; 2 Epidemiology Department, High Institute of Public Health, Alexandria University, Alexandria, Egypt; 3 Family Health Department- Mental Health Specialty, High Institute of Public Health, Alexandria University, Alexandria, Egypt; 4 Pharmacist, Egyptian Ministry of Health and Population, Alexandria, Egypt; 5 Department of Medical-Surgical Nursing, College of Nursing, Princess Nourah Bint Abdulrahman University, Riyadh, Saudi Arabia; 6 Family and Community Medicine Department, College of Medicine, King Khalid University, Abha, Saudi Arabia; 7 Tropical Health Department, High Institute of Public Health, Alexandria University, Alexandria, Egypt

**Keywords:** COVID-19, Vaccine confidence, Fully vaccinated, Validity, Reliability, Vaccine hesitancy, ARAB-VAX-CONF

## Abstract

**Background:**

Vaccine confidence is described as a belief that vaccines are effective, safe, and part of a trustworthy health system. The objective of this study was to develop and validate an Arabic tool to evaluate confidence in the received coronavirus disease 2019 (COVID-19) vaccines (ARAB-VAX-CONF).

**Methods:**

The research team developed the ARAB-VAX-CONF based on three areas specified by the Centers for Disease Control and Prevention (CDC): confidence in vaccine effectiveness, confidence in vaccine safety, and confidence in the healthcare system. The analysis includes data from 406 eligible vaccinated persons (≥ 18 years). Cronbach’s alpha was used to measure internal consistency, while convergent, discriminant, exploratory, and confirmatory factor analyses were used to verify construct validity.

**Results:**

A total of 406 adults were recruited, with a mean age of 33.0 ± 12.2 years. A total of 63.1% were males, 56.7% were married, 21.9% had chronic conditions, 93.3% were nonsmokers, and 39.6% were obligated to obtain vaccines. The three domains of the scale met the criterion of internal consistency (Cronbach’s alpha > 0.7). Convergent validity was identified by the significant inter-item and item-mean score of the domain correlation (*P* < 0.001). Discriminant validity was reported as the inter-factor correlation matrix (< 0.7). The Kaiser–Meyer–Olkin sampling adequacy measure was 0.80, and Bartlett’s sphericity test was significant (*P* < 0.001). Exploratory factor analysis indicated that the scale could be summarized into three factors. Confirmatory factor analysis confirmed the adequate psychometric properties and fit with observed data (root-mean-square error of approximation = 0.08, comparative fit index = 0.945, Tucker-Lewis index = 0.924, standardized root-mean-square residual = 0.086, normed fit index = 0.918, and goodness-of-fit index = 0.909).

**Conclusions:**

The ARAB-VAX-CONF developed in this study is valid and reliable for assessing confidence in vaccine effectiveness, safety, and confidence in the healthcare system. The ARAB-VAX-CONF can support decision-makers in addressing the gap in vaccine confidence among various populations.

**Supplementary Information:**

The online version contains supplementary material available at 10.1186/s42506-024-00174-8.

## Introduction

Since December 2019, and for more than 3 years, the world has been affected by the novel severe acute respiratory distress syndrome coronavirus 2 (SARS-CoV-2), the causative agent of the COVID-19 pandemic. By January 2024, more than 772 million confirmed cases and more than 7 million deaths were reported globally, with varying patterns of morbidity and mortality [1, 2]. Since the last quarter of 2020, many COVID-19 vaccines have been authorized by the World Health Organization (WHO) through the Emergency Use Listing (EUL) process; as of January 2024, 15 vaccines had received the EUL [3]. COVID-19 vaccination has been considered the key to individuals resuming their everyday activities and restoring normal travel and commercial movements. As of January 2024, almost 13 billion COVID-19 vaccines have been administered worldwide [4].

Egypt has launched a vaccination program in addition to implementing several public health and social measures to combat the pandemic [5]. A recent community-based survey clarified that approximately 44.3% of eligible individuals were fully immunized [6]. COVID-19 vaccines have been proven to be safe and effective in controlling the disease [7]. A study using mathematical modelling estimated that COVID-19 vaccination has saved 14.4 million lives worldwide [8]. However, it is insufficient given the ongoing viral mutation, particularly variants of concern (VOCs), which cause new waves of increases in cases and deaths. Despite the many doses administered, several issues have merged to undermine public confidence in vaccines, particularly breakthrough infections. Vaccine hesitancy can cause delays and interrupt research and delivery programs and, in certain cases, leading to disease outbreaks [9]. The most serious example is the 2003–2004 boycott of polio vaccination that led to a resurgence of the disease [10]. Egypt also experienced hesitancy regarding vaccination during the 2009 H1N1 pandemic, when roughly 27.7% of the imported doses remained unused, primarily due to fear of adverse outcomes following vaccination [11]. Recently, skepticism about the COVID-19 vaccine and concerns about its safety have been reported among adults and children [6, 12].

The United States (US) Centers for Disease Control and Prevention (CDC) defines vaccine confidence as the idea that vaccines work, are safe, and are part of a trustworthy health system [13]. There is a wide range of behavioral diversity across various individuals and populations regarding vaccine confidence, which extends from vaccine acceptance, intake, and promotion on one side to vaccine resistance and rejection on the other [14]. Public concerns about vaccine safety and efficacy are historical and go back to the first development of vaccines. Before the COVID-19 pandemic, there was a global decline in confidence, demand, and utilization among the public [15, 16] and even among healthcare personnel (HCP) [17]. Moreover, COVID-19 has negatively affected the ongoing compulsory vaccination program in developing and developed countries [18]. High confidence in vaccination programs is crucial to maintaining high coverage rates [19]. Guidelines recommending annual COVID-19 vaccine boosters, and dual vaccination of COVID-19 and flu vaccine strategies, particularly among high-risk groups [20], might be challenged by ill confidence from the previously vaccinated population.

Many tools have been developed to measure vaccine confidence, including the Vaccine Confidence Index (VCI) [21] and Oxford COVID-19 Vaccine Confidence and Complacency Scale, which covered the four factors: collective importance of a COVID-19 vaccine, beliefs that the COVID-19 vaccine will work, speed of vaccine development, and adverse effects [22]. Additionally, another tool is the US trust measure which is used to assess and monitor parental confidence in the vaccine system [23]. It covered the following constructs: “information environment,” “trust,” “HCP,” “attitudes and beliefs,” and “social norms.” However, most of them did not specifically address the confidence in the healthcare system that developed and/or administered the vaccine but assessed the confidence in the vaccine delivery system (23).

This study aimed to develop and validate the first Arabic tool to assess post-vaccination confidence in COVID-19 vaccines as defined by the CDC, confidence in vaccine safety, confidence in vaccine effectiveness, and confidence in the healthcare system, named the ARAB-VAX-CONF. We hypothesized that ARAB-VAX-CONF is a valid and reliable tool for the assessment of vaccine confidence, and it would support public health decision-makers in addressing the gap in vaccine confidence among various populations.

## Methods

### Study design

An anonymous, online cross-sectional study.

### Study setting

The developed tool was displayed online through Google Forms. Data was obtained from adult general population living in Egypt during the time of data collection, by providing the link of a self-administered questionnaire once without reminders. The link was shared through social media platforms (Facebook, WhatsApp, and Twitter) and emails. Each participant could only provide one response.

### Data collection tool

The developed tool included three main sections: the first was an informed consent that clarifies the aim of the study and ensures the anonymity and confidentiality of data. The second section included sociodemographic data on age, gender, education, occupation, healthcare profession, marital status, number of family members, the number of family members aged 60 years and above living within the same setting, household income, smoking status, and comorbidities (hypertension, respiratory disease, immunological disease, diabetes, cardiac disease, renal disease, malignancy, and any mental health problems). This part also asked about past COVID-19 infections, the period since the last infection, self-reported symptoms, and the severity of the disease. The final part covered vaccination statistics, including types, doses, side effects after getting vaccines, and the status and causes of obligation to vaccination, if any.

The third section involved the three domains of the ARAB-VAX-CONF tool that was developed after a thorough review of literature measuring confidence in the COVID-19 vaccine. Items discussing confidence in vaccine effectiveness and safety were adapted from the Oxford Confidence and Complacency Scale [22], vaccine confidence among parents [24], and others [17, 21, 23, 25–30].

### Tool translation

Ten items adapted from the mentioned tools were forward translated into Arabic, and other items were developed in Arabic.

### Construct identification

Several research team meetings were held to identify the constructs and develop a pool of items for each construct based on previous literature [17, 21–31]. Development of items to incorporate under each construct is as follows: The initial questionnaire had 27 items, 22 items for confidence in vaccine effectiveness and safety, and 5 items for confidence in the healthcare system.

### Expert evaluation (face & content validity)

An expert panel of four public health specialists (methodologist, healthcare professional, microbiologist, language professional, and tropical disease professional) reviewed the language clarity of the items and whether the identified items covered the described structures (face and content validity). After two sessions, the researchers agreed to delete 11 items from the final questionnaire, which consisted of 16 items.

### Final scale and score interpretation

The final scale before the psychometric assessment comprised 16 items covering 3 domains. The first domain contained eight items that assessed confidence in vaccine effectiveness. The second domain contained four items to assess trust in vaccination safety, while the third domain contained four items to assess confidence in the healthcare system. Each item was evaluated using a 5-point Likert scale: 1 — completely disagree; 2 — disagree; 3 — neutral; 4 — agree; and 5 — completely agree. The overall score ranged from 16 to 80. The mean scores of items within each domain were computed, with a higher mean score indicating more agreement in that category. After using confirmatory factorial analysis, 4 items were further excluded, and the scale included 12 items only (supplementary file  no. 1).

### Pilot study

Each author was instructed to collect at least five pilot responses to evaluate the time required to complete the questionnaire, the response rate, the language, and the clarity of the generated tool. The time needed to complete the questionnaire ranged from 6 to 10 min. The nonresponse rate was 20%. Minimum changes were made to increase the comprehensibility of sociodemographic questions. The pilot answers were omitted from the final study.

### Sample size

Based on the sample size guidelines of having 10 participants’ responses to each item for questionnaire validation (ratio 10:1), to create the questionnaire, we recruitted 160 individuals [32]. Moreover, a priori sample size calculation for the structural equation modelling (SEM) technique to perform confirmatory factor analysis (CFA) showed that a minimum sample of 156 is required, with the following variables: anticipated effect size = 0.1, statistical power = 0.8, 3 latent variables, observed variables = 10, and probability level = 0.05. It was raised to 200 to ensure valid and reliable estimates of factor loadings and model fit indices [33]. To compensate for the 20% non-response rate, the sample was raised to 240.

### Inclusion and exclusion criteria

Egyptian general adult population (≥ 18 years) fully vaccinated with COVID-19 vaccines were included. Participants were excluded if they refused to consent or were ineligible for immunization; moreover, patients with mental illnesses requiring treatment for more than 2 weeks were excluded.

### Sampling technique

Non-probability snowball and convenience sampling procedures were used, and the data were collected from January 1 to April 15, 2023.

### Statistical analysis

Quantitative factors were summarized as mean ± SD, whereas qualitative variables were reported as frequency and percentage. The average scores for each domain were calculated. Pearson’s correlation analysis was utilized to compute the inter-item and item-to-mean score of the sub-scale correlation [32].

### Reliability and item analysis

Cronbach’s alphas were determined for each domain to determine internal consistency. As a rule of thumb, a Cronbach’s alpha of > 0.70 to 0.80 is regarded as reasonable for a scale for research usage, and an alpha > 0.80 is considered extremely good [34].

### Construct validity

It is determined using criterion-related validity and structural (factorial) validity.

#### Criterion-related validity

Convergent and discriminant (divergent) validity were used to assess criterion-related validity. Convergent validity was determined by examining the inter-item and item-to-mean scores of the domain correlation. Discriminant validity was evaluated by calculating the factor correlation matrix of the three domains.

#### Factorial analysis validity

Factor analysis was conducted in two stages: exploratory factor analysis (EFA) and confirmatory factor analysis (CFA). We randomly divided the participants into two groups using the randomly selected cases function of SPSS. The data file was then divided into 160 participants for EFA and 246 participants for CFA. The EFA aims to find the major factor structures for the set of defined elements and determine the number of latent factors without making any assumptions about factor relationships. Before EFA, the Kaiser–Meyer–Olkin (KMO) sampling adequacy measure and the Bartlett sphericity test were performed. KMO statistics vary from 0 to 1, with values closer to 1 suggesting increased adequacy of the factor analysis (*KMO* ≥ 0.6 low adequacy, *KMO* ≥ 0.7 medium adequacy, *KMO* ≥ 0.8 high adequacy, *KMO* ≥ 0.9 very high adequacy). If the *p*-value of the Bartlett test is < 0.05, then factorial analysis can be used. The number of factors extracted is based on eigenvalues (> 1) and scree plot. To determine the type of rotation, we first ran EFA using the principal component analysis with an oblique direct (oblimin rotation) to calculate the inter-factor correlation. Discriminant validity was assessed if inter-factor correlation based on the factor correlation matrix was less than 0.7. The final EFA was performed using the principal component analysis with orthogonal Varimax rotation. In interpreting the output, we defined that each factor should have at least 3 items with high factor loadings of 0.4 and higher on the primary factor and minimal cross-loadings on any of the other factors (*a* < 0.3) to reduce overlap between domains [35].

#### Confirmatory factor analysis

The CFA included 246 participants and attempted to assess how well the factor structure found in the EFA suited the observed data. Specifically, we evaluated the convergent and discriminant validity of the components and model fit metrics using the SEM technique [33]. We employed model fit parameters such as *RMSEA* < 0.08, *CFI* > 0.9, Tucker-Lewis index (*TLI* > 0.9), *SRMR* ≤ 0.08, *NFI* > 0.9, *GFI* > 0.9, and *χ*^2^/df < 3 [35]. Convergent validity was determined when the average variance extracted (AVE) values for the various factors exceeded 0.5. Discriminant validity was proven if the square root of AVE is greater than the intercorrelation between the factors [33]. We used the Statistical Package for Social Science (SPSS) (version 25, Chicago, USA) to execute most of the analyses. Meanwhile, SPSS AMOS (version 26, Chicago, US) was used to conduct the CFA.

## Results

This study included 406 vaccinated adults. Their mean age was 33.0 ± 12.2 years, 63.1% were males and 56.7% were married, 73.6% lived in urban areas, 89.9% had university degrees, 52.7% were HCP, 21.9% had chronic diseases, and 93.3% were nonsmokers (Table 1).

**Table 1:** Sociodemographic characteristics of the participants included in the ARAB-VAX-CONF tool validation, January 1 to April 15, 2023 (*n* = 406)

Variables	*n* (%)
**Age (years)**	
Mean (SD)	33.0 (12.2)
Median [min, max]	32 [18, 77]
**Gender** Male Females	256 (63.1) 150 (36.9)
**Marital status**	
Married	230 (56.7)
Single	164 (40.4)
Widow	9 (2.2)
Divorced	3 (0.7)
**Residence**	
Urban	299 (73.6)
Rural	107 (26.4)
**Number of family members**	
Mean ± SD	5.1 ± 3.3
Median [min, max]	5.0 [0, 51]
**Number of family members aged ≥ 60**	
0	251 (61.8)
1	101 (24.9)
2	49 (12.1)
≥ 3	4 (1.2)
**Education**	
Graduate/postgraduate	365 (89.9)
Secondary-technical education	31 (7.6)
Less than secondary education	10 (2.5)
**Occupation**	
Clerk	222 (54.7)
Student	108 (26.6)
Not working	31 (7.6)
Craftsman	16 (3.9)
Retired	16 (3.9)
Hand worker	13 (3.2)
HCP	214 (52.7)
Physician	134 (62.6)
Pharmacist	54 (25.2)
Administrative	8 (3.7)
Nursing	7 (3.3)
Support services	5 (2.3)
Physiotherapy	4 (1.9)
Dentist	2 (0.9)
**Suffering from chronic disease (s)***#*	89 (21.9)
Hypertension	38 (9.4)
Respiratory disease	31 (7.6)
Immunological disease	21 (5.2)
Diabetes	20 (4.9)
Cardiac disease	12 (3)
Renal disease	10 (2.5)
Malignancy	7 (1.7)
**Smoking status**	
Current smoker	18 (4.4)
Ex-smoker	9 (2.2)

^#^Responses are not mutually exclusive

Based on self-reporting, 46.3% of the studied population had COVID-19 disease, 27.8% did not have COVID-19 infection, and 25.9% did not know. At the time of the study, 23.4% of all participants had self-reported having COVID-19 more than a year ago. However, among those infected, 92.6% did not require hospitalization, and 74.5% had mild symptoms, while 87.8% reported COVID-19 infection among their family members (Table 2).

**Table 2:** History of COVID-19 infection among participants included in the ARAB-VAX-CONF tool validation, January 1 to April 15, 2023, (*n* = 406)

Variables	*n* (%)^#^
Self-reported last COVID-19 infection*	
< 6 months	35 (8.6)
6–12 months	58 (14.3)
> 12 months	95 (23.4)
**Need hospitalization due to COVID-19 infection?**	
No	174 (42.9)
Severity of COVID-19*	
Mild symptoms	140 (34.5)
Moderate symptoms	38 (9.4)
Severe symptoms	6 (1.5)
Need hospital admission	3 (0.7)
Need intensive care unit admission	1 (0.2)
Family member (s) infected with COVID-19*	
Yes	165 (40.6)
No	15 (3.7)
I don’t know	8 (2.0)
**Family member died due to COVID-19**	
Yes	21 (5.17)

^#^Percent was calculated from a total of 406

*Number of responses = 188

The most used vaccines were AstraZeneca (30.8%), Pfizer (22.7%), and Sinopharm (14.3%). A total of 42.6% of the sample received booster doses, and 39.6% were obligated to receive the vaccine, and the most common cause of obligation was job requirement. The most reported side effects were pain at the site of injection (28.1%), fever (24.5%), and flu-like symptoms (23.6%) (Table 3).

**Table 3:** COVID-19 vaccinations among the participants included in the ARAB-VAX-CONF tool validation, January 1 to April 15, 2023 (*n* = 406)

Variables	*n* (%)	
**Received COVID-19 vaccines**	**406 (100)**	
Primary series of vaccination	233 (57.4)	
Booster	173 (42.6)	
**Obligated to receive vaccines**	**161 (39.6)**	
***Causes of obligation#***		
Job requirement	103 (25.4)	
Travel	45 (11.1)	
Family pressure	17 (4.2)	
University requirements	7 (1.7)	
Peer pressure	4 (1)	
Entry to governmental facilities	4 (1)	
**Type of vaccine (doses received (main & booster)***#*		
AstraZeneca		164 (30.8)
Pfizer		121 (22.7)
Sinopharm		76 (14.3)
Sinovac		69 (13)
Johnson & Johnson		36 (6.8)
Moderna		32 (6.0)
I don’t know		25 (4.7)
Sputnik		9 (1.7)
**Side effects***#*		
No side effects		135 (24.5)
Pain at the site of injection		155 (28.1)
Fever		130 (23.6)
Flu-like symptoms		98 (17.8)
Bone pain		7 (1.3)
Allergy		6 (1.1)
Headache		5 (0.9)
Myalgia		3 (0.5)
Others≈		13 (2.6)

^#^Responses are not mutually exclusive

^≈^Others include bone and joint pain, cardiac symptoms, chest pain, difficulty breathing, drowsiness, hypertension, immediate fainting, joint pain, neck pain, neuralgia, severe abdominal pain, tachycardia, palpitations, bleeding, and weakness

The internal consistency of each domain was assessed. The Cronbach’s alpha for confidence in vaccine effectiveness was 0.854, confidence in vaccine safety was 0.783, and confidence in the healthcare system was 0.925. Convergent validity was measured using inter-mean score correlation matrix which shows the confidence domains in vaccine effectiveness, the inter-mean score correlation between each item and the domain ranged from 0.756 to 0.837, (*p* < 0.001), while for the domain measuring confidence in vaccine safety, the inter-mean score correlation between the domain and each item was 0.79, 0.85, and 0.86 for questions 2, 3, and 4, respectively, *p* < 0.001. The domain measuring confidence in the health system (0.90–0.94, *p* < 0.001) was strongly positively correlated with its related items, while divergent validity revealed that the domains had a low correlation with each other. For instance, the correlation between confidence in vaccine effectiveness and confidence in vaccine safety was 0.349 (*p* < 0.001), and confidence in vaccine safety and confidence in the healthcare system were 0.49 (*p* < 0.001). However, the correlation between confidence in vaccine effectiveness and confidence in the healthcare system was 0.62 (*p* < 0.001) (Table 4).

**Table 4:** Reliability and convergent validity testing of the ARAB-VAX-CONF tool domains and items

Domain (*n* = 160)	Mean ± SD	Inter-mean score correlation
***Confidence in vaccine effectiveness***		
q2_ec2	2.64 ± (1.24)	0.752 (< 0.001*)
q3_ec3	3.36 ± (1.067)	0.792 (< 0.001*)
q4_ec4	3.26 ± (1.21)	0.817 (< 0.001*)
q6_ec6	3.77 ± (1.01)	0.820 (< 0.001*)
q7_ec7	3.71 ± (1.16)	0.775 (< 0.001*)
* Cronbach’s alpha*	***0.85***	
***Confidence in vaccine safety***		
q10_sc2	3.31 ± (1.08)	0.798 (< 0.001*)
q11_sc3	3.12 ± (1.31)	0.849 (< 0.001*)
q12_sc4	3.19 ± (1.20)	0.861 (< 0.001*)
* Cronbach’s alpha*	***0.78***	
***Confidence in health system***		
q13_hsc1	3.18 ± (1.27)	0.929 (< 0.001*)
q14_hsc2	3.18 ± (1.26)	0.958 (< 0.001*)
q15_hsc3	3.19 ± (1.21)	0.899 (< 0.001*)
q16_hsc4	3.20 ± (1.22)	0.931 (< 0.001*)
* Cronbach’s alpha*	***0.93***	

^*^Significant (*p* ≤ 0.05)

### *Structural (factorial) validity*

**Fig. 1:**
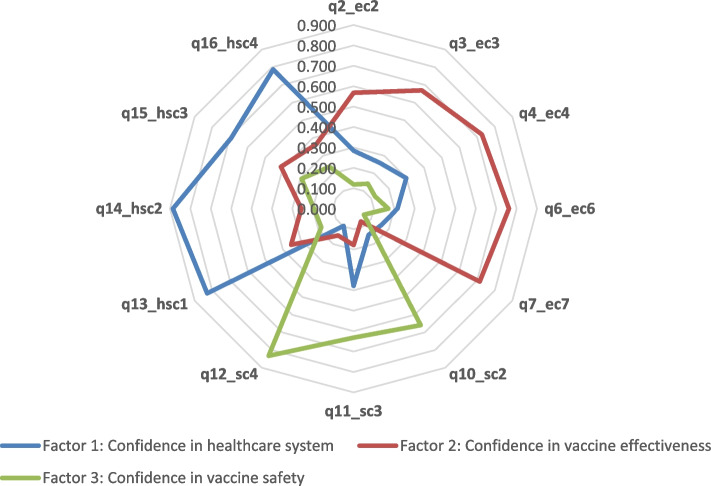
Exploratory factor analysis of the 12 questions related to the 3 domains of the ARAB-VAX-CONF tool

The KMO was 0.871, and the Bartlett’s sphericity test was significant (*p* < 0.001), which indicated the adequacy of the sample size. Additionally, all commonalities were ≥ 0.5. After using confirmatory factorial analysis, 4 items were further excluded, and the scale included 12 items only. The radar chart clarifies the loading of items on the three factors in the same manner as the questionnaire items (Fig. 1). The tools’ items addressing confidence in vaccine effectiveness are loaded with one factor and, similarly, items that address confidence in vaccine safety and items that address confidence in the healthcare system.

### Confirmatory factor analysis (CFA)

**Fig. 2:**
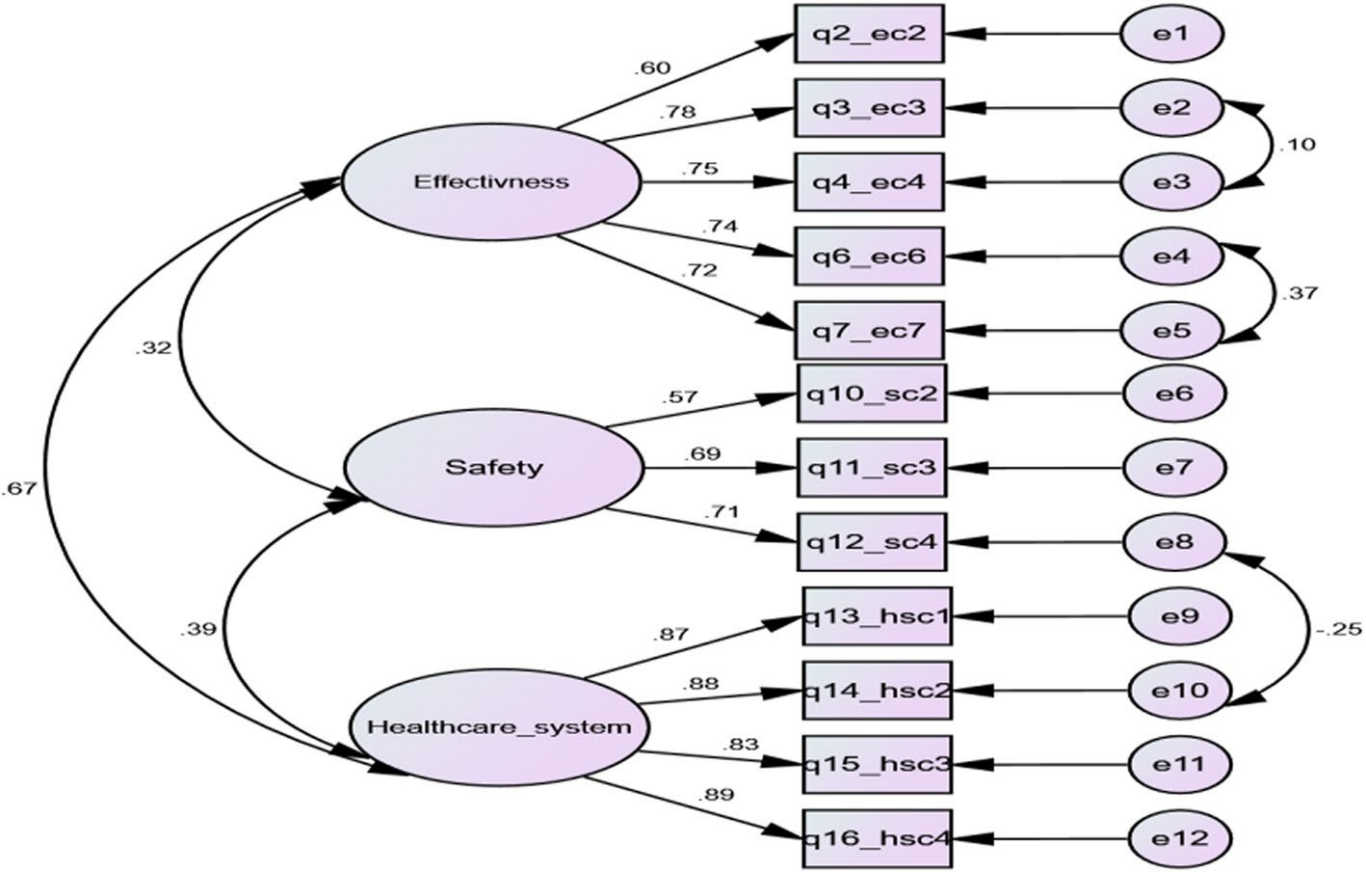
Confirmatory factor analysis of the 12 questions related to the 3 domains of the ARAB-VAX-CONF tool

The CFA revealed that all factor loadings ranged from 0.57 to 0.89. The construct reliability of the three factors was > 0.7. As for convergent validity, the AVE values were above 0.5 for the three factors. The correlation between the three latent variables was less than the square root of the AVE; hence, there was no problem with discriminant validity. The model fit parameters were *RMSEA* = 0.08, *CFI* = 0.945, *TLI* = 0.924, *SRMR* = 0.086, *NFI* = 0.918, and *GFI* = 0.909 (Fig. 2).

## Discussion

Controlling COVID-19 transmission has been extremely difficult, resulting in numerous ongoing attempts to develop effective vaccines. Vaccine hesitancy, whether for primary vaccinations or booster doses among adults and parents, remains a significant concern, rooted in a lack of trust in vaccines [36]. Another challenge was time, which led to the rapid development of COVID-19 vaccines through global collaboration. However, this rapid development, coupled with a lack of experimental studies, has harmed people’s trust and confidence in various vaccines manufactured by different laboratories. This highlights the importance of developing new tools, such as the one currently used, to assess true confidence in these new COVID-19 vaccines. However, among the few validated scales developed to address the confidence in the COVID-19 vaccine [21–23], none was valid for Arabic speakers or addressed the confidence of the three domains defined by the CDC. As a result, the current scale was created to assess confidence in previously developed COVID-19 vaccines, as understanding vaccine confidence is becoming increasingly important for public safety. Previously developed scales designed specifically for COVID-19 vaccines had the major limitation of focusing solely on one or two aspects of vaccine confidence rather than all three. As a result, our study contributes to these resources by providing a concise tool for assessing topics specific to our evolving discovery of individuals’ perspectives that influence COVID-19 vaccine confidence.

The present study was designed to create and validate a new scale that can be used to assess confidence in COVID-19 vaccination among Arabic speakers by addressing the three domains proposed by the CDC. The tool was primarily developed in 27 items which were further reduced to 16 items after expert evaluation. Furthermore, after psychometric evaluation, 4 items with inter-mean score correlation below 0.7 were removed from the tool. Finally, the validated tool involved 12 items: 5 items assessing confidence in vaccine effectiveness, 3 items assessing confidence in vaccine safety, and 4 items assessing confidence in the healthcare system.

Based on reliability analysis, Cronbach’s alpha for the three subscales was > 0.7 denoting acceptable internal consistency, with an inter-item correlation (0.3 – < 0.7). This was slightly lower than those obtained by the 14-item Oxford COVID-19 Vaccine Confidence and Complacency Scale developed by Freeman et al. [22]. Oxford scale was conducted with the participation of 5114 United Kingdom (UK) adults with a reliable Cronbach’s alpha (0.937) and a higher inter-factor correlation (0.82) [22]. The 7-item self-report COVID-19 Vaccine Concern Scale, developed by Forster et al., (2017), aimed to understand people’s fears and concerns about COVID-19 vaccines in a convenience sample of 2281 emergency medical service providers. It also had a slightly higher internal consistency (Cronbach’s alpha = 0.89) [37]. This slight difference in reliability values might be explained by the different contexts in which the scales were used. The CFA proved that all items were loaded on the relevant domain, with loadings ranging from 0.57 to 0.89. Hence, the questionnaire had good psychometric properties, and the model fits the observed data.

Most of the questions in our scale were designed to measure confidence in the effectiveness of the vaccine in preventing infection, which would significantly impact many preventive procedures and daily activities influenced by fear of infection. In return, this would help healthcare authorities to develop more effective public health strategies against the COVID-19 pandemic, helping to return to daily activities and stopping confinement which has several negative mental health consequences, such as depression, stress, anxiety, and fear [38]. Many people had moved away from their families and other loved ones, while others stopped properly using personal protective equipment, especially masks, due to its dermatological problems, including mainly acne, contact dermatitis, ulcers, and erosions [39]; our new scale was designed at a time when understanding vaccine confidence is becoming increasingly important to public safety.

Validating a tool for assessing confidence in COVID-19 vaccines would provide insight into issues that may influence an individual’s or community’s confidence in the COVID-19 vaccine. As a result, addressing these concerns about COVID-19 vaccines through targeted messaging or education, informed by efforts to understand vaccine confidence, has the potential to increase COVID-19 vaccination rates. Furthermore, it can contribute to herd immunity by lowering the virus’s prevalence and transmission in the community. It can also alleviate the strain on overburdened healthcare systems by lowering the number of severely ill COVID-19 patients.

### Strengths and limitations

One of the study’s strengths is that it is the first to develop and validate an Arabic scale for assessing the general population’s confidence in COVID-19 vaccinations.

We recognize that the current study has a few limitations that must be noted when interpreting its findings and should be addressed in future research. First, the use of a web-based survey to recruit eligible participants may have increased the likelihood of selection or no-response bias. However, during the pandemic, when restrictions were still in place, this recruitment method was the only viable option. Second, the initial questionnaire involved 27 questions, and 11 items were removed based on the agreement between experts, rather than calculating the content validity index. Third, the study included many HCP because they were among the first to get vaccinated, and our criteria required all participants to be fully vaccinated. Last, because the study was only conducted in Egypt, the results can only be projected to populations with similar characteristics. Other countries, including Arab ones, may require a reassessment of the developed scale with cultural modifications to maintain its effectiveness. Therefore, future research is necessary to assess its validity in other countries and languages.

## Conclusions

This study provides evidence for the adequate validity and reliability of the new Arabic COVID-19 vaccine confidence scale (ARAB-VAX-CONF). This could be very useful in determining the reasons behind confidence in vaccination, which would help propose adequate and effective strategies to advance vaccination coverage rates.

## Supplementary information

Supplementary Material 1

## Data Availability

The authors confirm that the data supporting the findings of this study are available within the article and its supplementary materials.

## Abbreviations

ARAB-VAX-CONF: Arabic tool for assessment of postvaccination confidence in COVID-19 vaccines
AVE: Average variance extracted
CDC: Centers of Disease Control and Prevention
CFA: Confirmation factor analysis
CFI: Comparative fit index
COVID-19: Coronavirus disease of 2019
EFA: Exploratory factor analysis
EUL: Emergency use listing
GFI: Goodness-of-fit index
HCP: Healthcare personnel
KMO: Kaiser-Meyer-Olkin
NFI: Normed fit index
RMSEA: Root-mean-square error of approximation
SD: Standard deviation
SEM: Structural equation modelling
SPSS AMOS: Statistical Package for the Social Sciences-Analysis of Moment Structures
SRMR: Standardized root-mean-square residual
TLI: Tucker-Lewis index
VCI: Vaccine Confidence Index
WHO: World Health Organization
