# Supplementary material for: Development and validation of an Arabic tool for assessment of post-vaccination confidence in COVID-19 vaccines (ARAB-VAX-CONF)
Source: J Egypt Public Health Assoc. 2024 Nov 18;99:28. doi: 10.1186/s42506-024-00174-8 (PMC11570568; doi:10.1186/s42506-024-00174-8)
Supplement: Supplementary file 1 — Supplementary Material 1 [file 42506_2024_174_MOESM1_ESM.pdf]

Tool for Assessment of Post Vaccination Confidence in COVID 19 Vaccines (ARAB-VAX-CONF)

آداة تقييم الثقة في تطعيم كوفيد ١٩

Authors: Rowan Abuyadek, Samar Abd ElHafeez , Mohamed Mostafa Tahoun, Sally Samir Othman , Abdelrahman Omran, Naglaa Fathy, Ramy Mohamed Ghazy

Thank you for choosing **(ARAB-VAX-CONF)** before you proceed, please take a moment to review and comply with the following terms of use:

Users are required to obtain an explicit permission from the authors before using the tool for any purpose beyond the personal use. To request permission, please contact: Rowan Abuyadek (rowan.abuyadek@alexu.edu.eg). Moreover, if you are using it in academic publications, please provide a citation to the original work.

الموافقة على المشاركة في البحث:

يهديكم فريق البحث أطيب تحية و يدعوكم للمشاركة في ملء هذا الأستبيان و الذي يهدف الي تقييم الثقة في لقاحات (تطعيمات) كوفيد 19 .

ملحوظة: هذا الأستبيان لن يطلب اي معلومات تدل على هويتك و لن يتم استخدام البيانات سوى لأغراض البحث. هل توافق على المشاركة في ملء هذا الأستبيان؟

(0) لا
(1) نعم

البيانات الاساسية:

1) النوع: (1) ذكر (2) أنثى
2) العمر:......................
3) الحالة الإجتماعية: (1) غير متزوج (2) متزج (2)أرمل (3) مطلق
4) عدد أفراد الأسرة:..................
5) عدد الأفراد فوق 60 سنه بالأسرة :.................
6) مستوى التعليم: (1) أمي/ يقرأ و يكتب (2) تعليم إبتدائى (3) تعليم إعدادى (4) تعليم ثانوى/حرفى (5) تعليم جامعى
7) الحالة الوظيفية: (0) لا يعمل (1) طالب (2) موظف (3) معاش (4) عامل يدوى (5) عامل حرفى
8) اذا كانت بالقطاع الطبى، فوظيفتك؟ (0) لا ينطبق (1) طبيب بشرى (2) صيدلى (3) طبيب اسنان (4) تمريض (5) علاج طبيعى (6) ادارى (7) خدمات مساعدة
9) دخل الأسرة: (1) لا يكفى ويستدين (2)لا يكفى (3) يكفى فقط (4) يكفى ويدخر
10) هل تعاني من اى امراض مزمنة؟ (0) لا (1) ضغط دم مرتفع (2) سكر (3) أمراض القلب (4) أمراض الكلى (5) الأورام (6) أمراض الجهاز التنفسي (7) أمراض جهاز المناعة (8) أخر تذكر
11) هل تدخن؟ (1) مدخن حالى (2) مدخن سابق (3)غير مدخن
12) هل تعاني من اى امراض نفسية؟ (0) لا (1) الاكتئاب (2) القلق (3) الوسواس القهرى (4) الفصام (5) أخرى تذكر

13) هل اصيبت بفيروس الكورونا؟ (0) لا (1) نعم (2) لا أعلم

14) اذا كانت الاجابة ب"نعم" عدد مرات الاصابة بفيروس الكورونا؟....................................

15) اخر اصابة بالكورونا كانت منذ: (1) أقل من 6 أشهر (2) 6-12 أشهر (3) أكثر من سنة

16) كيف تاكدت من الاصابة؟ (1) الاعراض (2) التحاليل (3) الاشعة
(يمكن اختيار اكثر من اجابة)

17) الاعراض التى اصيبت بها : (1) لا يوجد اعراض (2) فقدان حاسة التذوق
(يمكن اختيار اكثر من اجابة) (3) فقدان حاسة الشم (4) الحرارة
(5) ضعف بالعضلات (6) ضيق بالصدر
(7) الكحة (8) صعوبة فى التنفس
(9) الرعشة (10) أخرى تذكر

18) هل اصيب احد افراد العائلة بالكورونا؟ (0) لا (1) نعم (2) لا أعلم

19) هل توفى احد افراد الاسرة متأثرا بالكورونا؟ (0) لا (1) نعم (2) لا أعلم

20) هل أحتجت دخول المستشفى للعلاج من الكورونا؟ (0) لا نعم (1)

21) كيف تقييم شدة أعراض الإصابة (1) أعراض بسيطة (2) أعراض متوسطة (3) أعراض شديدة
(4) أحتجت لدخول مستشفى لتلقي العلاج (5) أحتجت لدخول العناية المركزة لتلقي العلاج

22) نوع التطعيم (أذكر اسم التطعيم): (0) غير مطعم (1) الجرعة الأولى:.........
(2) الجرعة الثانية:......... (3) الجرعة الثالثة:.........

23) هل تعرضت لاى اعراض جانبية (1) غير مطعم (2) لا
بسبب التطعيم؟ (3) الم مكان حقنة التطعيم (4) حرارة
(5) اعراض دور البرد (6) حساسية

24) هل تم اجبارك على تلقى التطعيم؟ (0) غير مطعم (1) ضغط من الاهل
(2) ضغط من الاصدقاء (3) اجبار العمل
(4) اغراض السفر (5) أخرى تذكر
(6) غير مطعم

25) في اي دولة تلقيت التطعيم :................ (0) غير مطعم

ثانياً: تقييم الثقة في تطعيم كوفيد ١٩							
مسلسل	كود	**- تقييم الثقة في فاعلية التطعيم**	اوافق بشدة	اوافق	محايد	لا اوافق	لا اوافق بشدة
١-	q2_ec2	تطعيم كوفيد ١٩ يحصن كل من أخذ التطعيم					
٢-	q3_ec3	تطعيم كوفيد ١٩ سيعزز جهازى المناعي بصورة كبيرة					
٣-	q4_ec4	تطعيم كوفيد ١٩سيعطيني الحرية في عودة الحياة الطبيعية					
٤-	q6_ec6	اذا تلقى الاخرون تطعيم كوفيد ١٩ فذلك سيحمي العديد من الارواح					
٥-	q7_ec7	توجد خطورة اذا لم يتلق أغلبية افراد المجتمع تطعيم كوفيد ١٩					
		**- تقييم الثقة في سلامة التطعيم**	اوافق بشدة	اوافق	محايد	لا اوافق	لا اوافق بشدة
٦-	q10_sc2	الاعراض الجانبية لتلقي تطعيمات كوفيد ١٩ خطيرة					
٧-	q11_sc3	تلقي تطعيمات كوفيد ١٩ تشعرني اني حقل تجارب					
٨-	q12_sc4	تطعيم كوفيد ١٩ يحمل مخاطر اكبر من التطعيمات الاخرى					
		**- تقييم الثقة في النظام الصحي**	اوافق بشدة	اوافق	محايد	لا اوافق	لا اوافق بشدة
٩-	q13_hsc1	اثق في المعلومات التي احصل عليها عن تطعيمات كوفيد ١٩					
١٠-	q14_hsc2	المعلومات التي اتلقاها عن تطعيمات كوفيد ١٩ من المنظومة الصحية جديرة بالثقة					
١١-	q15_hsc3	انا اثق في النظام الصحي الذي طرح تطعيم كوفيد ١٩ الذي تلقيتة					
١٢-	q16_hsc4	انا اثق في تطعيمات كوفيد ١٩ التي توفرها المنظومة الصحية في مجتمعي					
